# Supplementary material for: Development of management indicators of nursing for minimizing physical restraints focused on older adult patients hospitalized in acute care settings: A Delphi consensus study
Source: PLoS One. 2024 Jul 10;19(7):e0306920. doi: 10.1371/journal.pone.0306920 (PMC11236117; doi:10.1371/journal.pone.0306920)
Supplement: S1 Appendix — (DOCX) [file pone.0306920.s002.docx]

# S2 Appendix: Expert Panel Assessment of Indicators in the 1st to 3rd Rounds

# S2 Table 1. Top Management Indicator Evaluations in the 1st to 3rd Rounds

|  | **Draft indicator** | **1st round modified indicator (n = 23)** | **1–3 (n)** | **7–9 (n)** | **Median** | **2nd round modified indicator (n = 14)** | **1–3 (n)** | **7–9 (n)** | **Median** | **3rd round modified indicator (n = 12)** | **1–3 (n)** | **7–9 (n)** | **Median** |  |
| --- | --- | --- | --- | --- | --- | --- | --- | --- | --- | --- | --- | --- | --- | --- |
| **Planning** | | | | | | | | | | | | | | |
| 1 | Incorporating findings on minimizing physical restraints outside of hospitals and on the environment surrounding acute care hospitals | Gathering information on the environment surrounding acute care hospitals and findings on minimizing physical restraints outside of hospitals | 3 | 19 | 8 |  | 0 | 13 | 8 |  | 0 | 10 | 8 |  |
| 2 | Visualization of issues related to physical restraints occurring in the nursing department | Visualization of issues related to physical restraints occurring in the field | 0 | 20 | 8 |  | 0 | 14 | 9 |  | 0 | 12 | 8.5 |  |
| 3 | Statement of policy to minimize physical restraints |  | 0 | 21 | 9 |  | 0 | 14 | 9 | Statement of policy to minimizing physical restraints at the hospital or nursing department | 0 | 12 | 9 |  |
| 4 | Instilling the policy of minimizing physical restraints at the nursing department |  | 0 | 22 | 9 |  | 0 | 14 | 9 | Spreading the policy of minimizing physical restraints throughout the nursing department | 0 | 12 | 9 |  |
| 5 | Clarification on the definition of physical restraint |  | 0 | 19 | 9 |  | 0 | 13 | 9 |  | 0 | 12 | 9 |  |
| 6 | Providing standards in the hospital to determine the need for physical restraints | Providing reference standards in the hospital to determine the need for physical restraints | 0 | 19 | 9 |  | 0 | 13 | 9 |  | 0 | 12 | 9 |  |
| 7 | Creating manuals or guidelines that address the factors that lead to the implementation of physical restraints |  | 2 | 21 | 8 | Utilization of existing manuals or guidelines or creating ones that address the factors that lead to the implementation of physical restraints | 0 | 13 | 9 | Utilization of existing manuals or guidelines or creating ones that address the factors that lead to the implementation of physical restraints at each hospital | 0 | 12 | 9 |  |
| **Motivating** | | | | | | | | | | | | | |  |
| 8 | Sharing success experiences of minimizing physical restraints within the nursing department |  | 1 | 20 | 8 |  | 0 | 11 | 9 |  | 0 | 12 | 9 |  |
| 9 | Sharing the reactions of patients and families to the implementation of nursing care to minimize physical restraints within the nursing department |  | 2 | 18 | 8 |  | 1 | 12 | 9 |  | 0 | 12 | 8.5 |  |
| 10 | Being an organizational culture to reduce physical restraints | Creating an organizational culture with daily initiatives and discussions toward minimizing physical restraints | 0 | 21 | 9 |  | 0 | 12 | 9 | Attempting to create an organizational culture with daily initiatives and discussions toward minimizing physical restraints | 0 | 12 | 9 |  |
| **Training** | | | | | | | | | | | | | |  |
| 11 | Planning for required education to minimize physical restraints based on the actual situation and existing evidence of physical restraints |  | 1 | 18 | 8 | Planning for required education to minimize physical restraints concerning the actual situation and existing evidence of physical restraints | 0 | 11 | 9 |  | 0 | 12 | 8 |  |
| 12 | Assigning staff in charge of promoting the minimization of physical restraints at each department | Planning to develop leaders who will respect the dignity of patients and improve their ethical competence | 3 | 15 | 8 |  | 0 | 14 | 9 |  | 0 | 11 | 9 |  |
| 13 | Educating the staff to spread the correct knowledge and skills necessary for minimizing physical restraints | Educating the staff to spread the correct knowledge and skills necessary to not accept physical restraints | 1 | 17 | 8 | Providing opportunities to spread the correct knowledge and skills necessary to not accept physical restraints | 1 | 12 | 8 | Providing opportunities to spread the correct knowledge and skills necessary to not implement physical restraint whenever possible | 1 | 11 | 9 |  |
| 14 | Educating the staff to acquire skills to minimize physical restraints | Maintenance of an educational system for basic older adult patients’ care and delirium care | 1 | 16 | 8 |  | 0 | 13 | 9 |  | 0 | 12 | 9 |  |
| 15 | Researching efforts to minimize physical restraints | Recommending research-based efforts to minimize physical restraints | 3 | 15 | 7 |  | 0 | 13 | 8 |  | 0 | 10 | 8 |  |
| 16 | Adopting educational tools to ensure that all staff members receive education related to minimizing physical restraints | Adopting educational tools to ensure that all staff members receive necessary education regard of minimizing physical restraints | 1 | 16 | 8 |  | 0 | 13 | 9 | Adopting educational tools to ensure that all staff members receive necessary education for minimizing physical restraints | 1 | 11 | 8 |  |
| 17 | Encouraging staff voluntary growth and helping it reflect on daily ethics and usual nursing practice regarding physical restraints from an ethical perspective | Recommend creating opportunities to reflect on daily ethics and usual nursing practice with a focus on patient intention and quality of life | 1 | 18 | 8 |  | 0 | 13 | 9 |  | 0 | 12 | 9 |  |
| **Commanding** | | | | | | | | | | | | | |  |
| 18 | Survey and analysis of staff’s awareness of physical restraints | Confirming staff’s awareness to respect the dignity of patients | 2 | 14 | 8 | Confirming the staff’s awareness of respect for dignity as perceived through daily interactions | 1 | 11 | 8 | Confirming the staff’s feelings and awareness of respect for dignity in minimizing physical restraints, as perceived through daily interactions | 0 | 11 | 8 |  |
| 19 | Sharing with each department the discussions among managers and committees regarding physical restraints to review ethics aspect | Sharing with each department the discussions and measures among managers and committees regarding physical restraints | 0 | 19 | 8 | Sharing with each department the discussions and improvement measures among managers and committees regarding physical restraints | 1 | 10 | 8 |  | 0 | 12 | 8.5 |  |
| 20 | Intentionally involving individuals in a leadership position in the department: the head nurse and chief nurse | Proactively interacting with individuals in a leadership position in the department: the head nurse and chief nurse | 0 | 20 | 9 | Proactively interacting with individuals in a leadership position in the department: the head nurse and chief nurse, and involving them in efforts to minimize physical restraints | 0 | 10 | 9 |  | 0 | 12 | 9 |  |
| 21 | Considering the impact of no use of physical restraints on hospital management and sharing with other departments and nursing departments | Supporting each department’s efforts to minimize physical restraints from a managerial perspective, and managing the hospital or nursing departments | 4 | 15 | 8 | Supporting each department’s efforts to minimize physical restraints from a managerial perspective, and participating in the management of hospital and nursing departments | 1 | 11 | 8 | Supporting each department’s efforts to minimize physical restraints from a managerial perspective, and discussing the direction with the organization’s executive managers | 0 | 10 | 8 |  |
| **Organizing** | | | | | | | | | | | | | |  |
| 22 | Placing cross-organizational teams to promote minimizing physical restraints | Utilizing committees and teams to promote minimizing physical restraints | 2 | 18 | 8 |  | 0 | 13 | 9 |  | 0 | 12 | 9 |  |
| 23 | Making efforts to minimize physical restraints by multidisciplinary teams or multiple staff | Supporting efforts to minimize physical restraints by multidisciplinary teams | 0 | 18 | 8 |  | 0 | 12 | 9 |  | 0 | 12 | 9 |  |
| 24 | Considering care methods to cooperate with staff in each department and cross-sectional teams |  | 1 | 19 | 8 |  | 0 | 13 | 8 |  | 0 | 11 | 8 |  |
| 25 | Creating a system to consult with specialists in the hospital regarding physical restraints |  | 1 | 20 | 8 |  | 0 | 13 | 9 |  | 0 | 11 | 8.5 |  |
| 26 | Did not exist | [create a new item] Creating a mechanism to consult with teams or specialists in the hospital when ethical issues arise |  |  |  |  | 0 | 14 | 9 |  | 0 | 11 | 9 |  |
| 27 | Establishing a system to share ethical issues related to physical restraints with managers, each committee, and each medical department | Sharing ethical issues related to physical restraints with other departments, and establishing a system of collaboration that allows multidisciplinary dialogue | 3 | 18 | 8 |  | 0 | 13 | 8 | Sharing ethical issues related to physical restraints with other departments, including each medical department, and establishing a system of collaboration that allows multidisciplinary dialogue | 0 | 11 | 8 |  |
| 28 | Devising a work system to look after patients | Establishing a flexible cross-departmental support system to ensure that staff are available to look after patients | 1 | 20 | 8 |  | 0 | 11 | 8 |  | 0 | 11 | 8 |  |
| 29 | Showing attitude to guarantee responsibility against accidents associated with minimizing physical restraints | Showing attitude to guarantee organization responsibility against accidents associated with minimizing physical restraints | 3 | 17 | 9 |  | 0 | 13 | 8.5 | Guaranteeing organization responsibility against accidents associated with minimizing physical restraints | 0 | 11 | 9 |  |
| 30 | Did not exist | [creating a new item] Creating mechanisms and opportunities for patients and families to understand the minimizing physical restraints as an organization |  |  |  |  | 1 | 11 | 8 |  | 0 | 12 | 8 |  |
| 31 | Creating an environment, maintenance, and management of supplies to prevent accidents |  | 1 | 19 | 8 |  | **0** | **13** | 8 |  | **0** | **12** | 8.5 |  |
| **Controlling** | | | | | | | | | | | | | |  |
| 32 | Ongoing evaluation of the efforts progress minimizing physical restraints at your hospital |  | 1 | 19 | 8 |  | 0 | 14 | 9 |  | 0 | 12 | 9 |  |
| 33 | Survey and analysis of the number and percentage of physical restraints in the hospital | Periodic survey and analysis of indicators such as the number and percentage of physical restraints used and fall rates | 0 | 21 | 8 |  | 0 | 12 | 8 |  | 0 | 12 | 8.5 |  |
| 34 | Analysis of physical restraint rates by comparing with external evaluation criteria |  | 1 | 17 | 8 |  | 1 | 11 | 8 | Analysis of physical restraint rates with reference to external evaluation criteria | 0 | 11 | 7 |  |
| 35 | Reflecting for practice from the results of the survey analysis for physical restraints | Sharing the results of the survey for physical restraints and discussing improvement measures together with all staff | 1 | 17 | 8 | Identifying issues in work processes based on the results of the survey analysis for physical restraints and discussing improvement measures together with each department | 2 | 10 | 7.5 |  | 0 | 10 | 8 |  |
| **Delete indicators** | | | | | | | | | | | | | |  |
| 25 | Placing a committee to review issues related to physical restraints | [delete] (integrating with No. 23) | 3 | 17 | 7 |  |  |  |  |  |  |  |  |  |
| 29 | Creating opportunities to reflect on nursing practices from the patient’s perspective to foster an ethical view of physical restraints |  | 1 | 19 | 8 | [delete] (integrating to No. 14) | 0 | 11 | 8.5 |  |  |  |  |  |

# S2 Table 2. Middle Management Indicator Evaluations in the 1st to 3rd Rounds

|  | **Draft indicator** | **1st round modified indicator (n = 23)** | **1–3 (n)** | **7–9 (n)** | **Median** | **2nd round modified indicator (n = 13)** | **1–3 (n)** | **7–9 (n)** | **Median** | **3rd round modified indicator (n = 13)** | **1–3 (n)** | **7–9 (n)** | **Median** |
| --- | --- | --- | --- | --- | --- | --- | --- | --- | --- | --- | --- | --- | --- |
| **Planning** | | | | | | | | | | | | | |
| 1 | Visualization of issues related to physical restraints in the organization | Visualization of issues related to physical restraints in the department | 1 | 18 | 8 |  | 0 | 12 | 9 |  | 0 | 12 | 9 |
| 2 | Developing departmental targets to understand the nursing department’s policy to minimize physical restraints | Developing and penetrating departmental targets to understand the nursing department’s policy to minimize physical restraints | 1 | 20 | 8 |  | 0 | 13 | 9 | Developing and spreading departmental targets to understand the nursing department’s policy to minimize physical restraints | 0 | 13 | 9 |
| 3 | Documenting standards for staff to determine the need for physical restraints | Providing reference standards to determine the need for physical restraints that staff can refer | 1 | 16 | 8 |  | 0 | 12 | 9 |  | 0 | 12 | 8 |
| 4 | Grasping the status of efforts to minimize physical restraints | Grasping the status of efforts to minimize physical restraints based on committee and departmental discussions | 0 | 20 | 9 |  | 0 | 13 | 9 |  | 0 | 12 | 9 |
| 5 | Encouraging staff to take advantage of problem-solving opportunities with multidisciplinary to minimize physical restraints | Encouraging staff to participate in discussions with physicians and other professionals to minimize physical restraints | 0 | 19 | 8 | Striving to participate in discussions with physicians and other professionals to minimize physical restraints, and understanding the results of these discussions | 0 | 9 | 9 |  | 0 | 12 | 8 |
| **Motivating** | | | | | | | | | | | | | |
| 6 | Admitting staff’s positive attitude toward minimizing physical restraints | Positive evaluation of staff’s positive attitude toward minimizing physical restraints | 0 | 22 | 9 |  | 0 | 12 | 9 |  | 0 | 13 | 9 |
| 7 | Did not exist | [creating a new item]  Creating an organizational culture that fosters efforts and discussions to minimize the physical restraint for staff on a daily basis |  |  |  | Feedback recognizing the good points and autonomy of efforts to minimize physical restraints for staff on a daily basis | 0 | 12 | 9 |  | 0 | 12 | 9 |
| 8 | Feedback on what is discussed about physical restraints at committee meetings and in administrative departments for staff |  | 0 | 19 | 9 |  | 0 | 13 | 8 |  | 0 | 12 | 8 |
| 9 | Improving staff self-efficacy by sharing patient/family responses to staff who have implemented nursing care to minimize physical restraints |  | 1 | 19 | 9 |  | 1 | 12 | 9 |  | 0 | 12 | 9 |
| 10 | Sharing success experiences of minimizing physical restraints | Creating opportunities for staff to share success experiences of minimizing physical restraints | 1 | 19 | 9 |  | 0 | 13 | 9 |  | 0 | 13 | 9 |
| **Training** | | | | | | | | | | | | | |
| 11 | Providing opportunities or encouraging participation in education to spread the correct knowledge and skills about physical restraints | Providing opportunities or encouraging participation in education to spread the correct knowledge and skills necessary to not accept physical restraints | 0 | 19 | 8 | Providing opportunities or encouraging participation in education to spread the correct knowledge and skills necessary to not implement physical restraint whenever possible | 1 | 10 | 8 |  | 0 | 13 | 9 |
| 12 | Providing opportunities or encouraging participation in education as a base for older adult patients’ care and delirium care | Providing opportunities or encouraging participation in education as a base for older adult patients’ care and delirium care | 0 | 18 | 8 |  | 0 | 12 | 9 |  | 0 | 13 | 9 |
| 13 | Enabling reflection on nursing care from the patient’s point of view to foster an ethical view of physical restraints | Setting up opportunities to reflect on nursing care from the patient’s point of view to foster an ethical view of physical restraints | 0 | 21 | 9 |  | 0 | 13 | 9 |  | 0 | 13 | 9 |
| 14 | Enhancing to reflect on usual nursing practice from the perspective of ethics | Enhancing to reflect on daily ethics and usual nursing practice with a focus on patient intention and quality of life | 1 | 20 | 8 | Providing opportunities to reflect on daily ethics and usual nursing practice with a focus on patient intention and quality of life | 0 | 11 | 9 |  | 0 | 13 | 9 |
| 15 | Research practice to minimize physical restraints | Research-based practice to minimize physical restraints | 5 | 15 | 7 | Active action for research-based practice and practice reporting about successful cases to minimize physical restraints | 2 | 9 | 7 |  | 0 | 11 | 8 |
| **Commanding** | | | | | | | | | | | | | |
| 16 | Setting up a discussion forum for multiple staff members in the department to discuss minimizing physical restraints |  | 1 | 20 | **9** |  | 0 | 12 | 9 |  | 0 | 13 | **9** |
| 17 | Providing opportunities for dialogue with patients and families regarding physical restraints to gain their understanding and cooperation in minimizing physical restraints |  | 1 | 16 | 7 |  | 1 | 12 | 9 |  | 1 | 12 | 8 |
| 18 | Considering care methods for factors that contribute to physical restraints with cross-functional teams and specialists |  | 1 | 19 | 8 |  | 0 | 13 | 8 |  | **0** | **12** | 9 |
| 19 | Discussing with other professionals and specialists about ethical dilemmas regarding physical restraints |  | 0 | 20 | 9 |  | 0 | 12 | 9 |  | 1 | 12 | 9 |
| 20 | Sharing ethical issues related to physical restraints with staff |  | 0 | 20 | 9 |  | 0 | 12 | 9 |  | 0 | 13 | 9 |
| 21 | Collaborating with staff to determine care alternatives to physical restraints in the field | Work with staff to determine care alternatives to physical restraints when staff members are struggling | 1 | 16 | 8 |  | 1 | 12 | 9 |  | 0 | 13 | 9 |
| 22 | Use of manuals and guidelines to address factors of physical restraints adopted | Use of manuals and guidelines to address factors of physical restraints adopted by the facility | 1 | 18 | 8 |  | 1 | 11 | 9 |  | 1 | 11 | 8 |
| 23 | Negotiation with administrative departments on providing necessary environmental arrangements to minimize physical restraints |  | 1 | 17 | 8 |  | 1 | 11 | 9 |  | 1 | 11 | 9 |
| **Organizing** | | | | | | | | | | | | | |
| 24 | Supporting the activities of staff that promote minimizing physical restraints |  | 1 | 20 | 9 |  | 0 | 13 | 9 |  | 0 | 13 | 8 |
| 25 | Trying to discuss the point of removal of physical restraints | Discussing alternatives to physical restraints and the practice of dealing with patients mainly by department leaders | 1 | 20 | 9 | Discussing led by department leaders about alternatives to physical restraints and the practice of dealing with patients | 0 | 13 | 9 |  | 0 | 11 | 9 |
| 26 | Responding to reduce fear about accidents associated with minimizing physical restraint with an attitude of accepting responsibility | Responding to reduce fear of accidents associated with minimizing physical restraint | 1 | 16 | 9 |  | 0 | 13 | 9 | Responding to prevent individual fear of accidents associated with minimizing physical restraint through team discussions | 0 | 12 | 9 |
| 27 | Prepare the accident prevention items and restraint substitutes | Maintenance of accident prevention items and restraint substitutes in department for use at all time | 1 | 18 | 9 | Maintenance of department-owned accident prevention items and restraint substitutes for available use when needed | 1 | 11 | **8** |  | 0 | 13 | 8 |
| **Controlling** | | | | | | | | | | | | | |
| 28 | Survey and analysis of the number and percentage of physical restraints | Survey and analysis of the number and percentage of physical restraints and indicators of falling and so on periodically | 1 | 19 | 8 |  | 0 | 12 | 9 |  | 1 | 12 | 8 |
| **Delete indicators** | | | | | | | | | | | | | |
|  | Communicating to staff the policy of minimizing physical restraints | [delete] (integrating with No. 2) | 1 | 20 | 9 |  |  |  |  |  |  |  |  |
|  | Recommending the use of educational tools to ensure that all staff receive education on physical restraints | [delete] (integrating Nos. 12 and 13) | 1 | 16 | 8 |  |  |  |  |  |  |  |  |
|  | Involving the chief and specialists to facilitate so that staff can discuss care of minimizing physical restraints | [delete] (integrating with No. 21) | 0 | 18 | 8 |  |  |  |  |  |  |  |  |
|  | Survey and analysis of staff’s awareness of physical restraints | [delete] (lack of consensus) | 3 | 13 | 7 |  |  |  |  |  |  |  |  |
|  | Analysis of physical restraint rates with comparing to external evaluation criteria | [delete] (lack of consensus) | 4 | 11 | 7 |  |  |  |  |  |  |  |  |
|  | Reflecting for practice from the results of the survey analysis for physical restraints | [delete] (lack of consensus) | 4 | 15 | 7 |  |  |  |  |  |  |  |  |

# S2 Table 3. Comments from Expert Panels for Top Management Indicators in the 1st to 3rd Rounds

|  | **1st round modified indicator (n = 23)** | **2nd round modified indicator (n = 14)** | **3rd round modified indicator (n = 12)** |
| --- | --- | --- | --- |
| **Planning** | | | |
| 1 |  |  | The effectiveness of the information collection is unknown since physical restraints are still common in acute care hospitals. |
| 2 | Carefully share nurses’ spontaneous ideas. |  |  |
| 3 | It is important to gain an understanding through dialogue and to approach each nurse’s desire not to restrain in the first place.  As a top nursing leader, it is absolutely necessary to express an unwavering will and policy. |  | As a hospital? As a nursing department? |
| 4 | It is important to gain an understanding through dialogue and to approach each nurse’s desire not to restrain in the first place. |  | Judging “instilling” is difficult. I think the expression overlaps with Nos. 3 and 6. |
| 5 | It is good if it is clearly stated somewhere in the organization (e.g., in a committee).  Necessary to some extent, but it is essential to be flexible and to include the target’s point of view. |  |  |
| 6 | It is good if it is clearly stated somewhere in the organization (e.g., in a committee).  Necessary to some extent, but it is essential to be flexible and include the target’s point of view. |  |  |
| 7 |  | We believe that it is effective to use guidelines that are already available to the public without having to create our own guidelines. | I think “at each hospital” is necessary. |
| **Motivating** | | | |
| 8 |  | I think the expression overlaps with No. 9. |  |
| 9 | It is necessary to create mechanisms and opportunities to gain an understanding of minimizing physical restraints with families rather than sharing reactions. Middle management indicator No. 17 is also needed as a top management indicator. |  |  |
| 10 | Hard to understand what the nursing management of “being an organizational culture” means. | Organizational culture is difficult to define. I wonder if organizational culture is something that can be created. | It is difficult to determine whether one can establish an organizational culture. I believe that this expression may overlap with others when evaluating it in terms of behavioral practices and discussions. |
| **Training** | | | |
| 11 |  | Even if it is evidence-based, I question whether the content is valid in clinical practice. | This indicator is appropriate because the required education varies by hospital function and department. |
| 12 |  |  |  |
| 13 | I think that dissemination of knowledge and technology is the role of those with expertise. | I believe that the use of the word “not to accept” demoralizes staff and does not lead to positive organizational change. | There are multiple factors, and knowledge does not mean that physical restraint is not an option. |
| 14 | I think that dissemination of knowledge and technology is the role of those with expertise. There is a lot to be educated by practitioners. I think education on base elder care and delirium care is more important than education on physical restraints. |  |  |
| 15 |  |  |  |
| 16 | “Education regard of minimizing physical restraints” is more suitable. |  | The content of this indicator is unclear compared with other “training” indicators. |
| 17 | I think it is important to focus on the patient’s wishes and quality of life instead of focusing on physical restraints and looking back ethically. |  |  |
| **Commanding** | | | |
| 18 | Research and analysis are necessary for management, but whether they are linked to nursing practice needs to be considered. I think it is good to do it naturally, casually, and occasionally. | The awareness survey is not invalid, but its appropriateness and effectiveness as an indicator is unclear. | Physical restraints occur even when respect for individual dignity is understood. It is necessary to be careful not to lead to staff dissatisfaction that they feel managers do not understand. It is unclear is this indicator is included in direct feedback to staff or not. |
| 19 | I think it is necessary to create a mechanism for ethics consultation within the hospital. | If the safety department receives a report on an incident due to the removal of physical restraints, it could still be considered necessary to use physical restraints. |  |
| 20 | Dialogue is needed. | It is hard to determine who is interacting with whom. |  |
| 21 | It is difficult at present to see the impact on hospital management as a numerical value. We would like to show that not using physical restraints is better for patients’ ADL and QOL and contributes to shorter hospital stays. | Nursing quality improvement often does not directly translate into management results. | The expression of 2nd round is acceptable. Participating in management is not always required, even to the point of supporting. Hospitals are affected by improvements in numerical values with the quality of medical care. |
| **Organizing** | | | |
| 22 | It is practically difficult to set up a team dedicated to minimizing physical restraints; that is, utilize existing teams, committees, and so on. | I think the expression overlaps with No. 24. |  |
| 23 | I think we need to operate as one team. There are some aspects that are difficult in departments in which patients’ awareness and cognitive functions are declining, such as in emergency medical services, and there are differences in efforts between departments. |  |  |
| 24 | Collaboration with physicians is vital. |  |  |
| 25 |  | I think the expression overlaps with No. 22 and 24. |  |
| 26 |  |  |  |
| 27 |  |  | Cooperation with physicians is essential for minimizing physical restraints. |
| 28 | I think it is necessary if it means a flexible support system. I think it is necessary to obtain the team’s view and support. It is important to secure human resources: for example, by utilizing relievers only to watch over the team. | I am concerned that the department relies on support from outside the department and has not developed response capacity within the department. |  |
| 29 | I think it is important to show how we guarantee responsibility and that is accepted by staff. I think it is important for the top management to guarantee responsibility. |  | “Guaranteeing” responsibility is more appropriate for nursing management practice. |
| 30 |  |  |  |
| 31 |  |  |  |
| **Controlling** | | | |
| 32 |  |  |  |
| 33 | Research and analysis is necessary for management, but whether it leads to nursing practice needs to be considered |  |  |
| 34 | Research and analysis is necessary for management, but whether it leads to nursing practice needs to be considered | It is not sufficient to end with an analysis. | “Reference” to external evaluation criteria is more appropriate because the method of calculating the physical restraint rate is not unified. |
| 35 | Research and analysis is necessary for management, but whether it leads to nursing practice needs to be considered | How does this translate into practice? | Examining each case that needed physical restraint is more important than identifying issues in the work process. This indicator is necessary in case of the preparations for physical restraint before patients’ assessment in work process. |
| **Delete indicators** | | | |
| 36 | It is practically difficult to set up a team dedicated to minimizing physical restraint. Utilize existing teams, committees, and so on? |  |  |
| 37 |  | I think the expression overlaps with No. 14. |  |
| **Free comment** | | | |
|  | Preparedness for nursing practice to not consider physical restraint as the first choice is important. | It is important to reach consensus concerning policy-related decisions with the head of the organization, such as the hospital director. Moreover, sharing the policy with the physicians is essential, as they are responsible for the medical treatment. | I agree that it is important to align with the head of the organization on policy-related decisions, with reference to the free comment in the 2nd round. |
|  | I am glad if nurse executives practice at least one indicator from Nos. 1‒35. | It is good to promote initiatives to create groups that action the philosophy of the nursing top management. | It is important that the medical safety committee collaborate with the dementia care team and the delirium care team to enhance quality of care |
|  | Statement of policy and vision from the head of the nursing department is important. | As physical restraints are performed under the direction of a doctor, effective engagement of nursing managers with doctors could promote better nursing practice. | Clarification of the cause of incidents and accidents that occur after the removal of physical restraints is needed. |
|  | Minimization of physical restraints should be practiced by the whole organization and not just by nurses. Cooperation with physicians is essential for minimizing physical restraints. Showing attitude and discussing to gain understanding and cooperation from other departments as the head of the nursing department is important. | Understanding each patient’s needs and providing appropriate care has led to nursing care not relying on physical restraints. We shared this achievement with the field, which has led to the minimization of physical restraints. | For each item, I commented on whether it is helpful to clarify the wording of whether it is direct practice or coordination by top managers. |
|  | I think that it is essential for the nursing top management to support head nurses, teams, and committees in minimizing physical restraints. | It could be necessary for the organization to have a set method of handling incident reports in care practices aimed at minimizing physical restraints, including the policy of the medical safety team. | It is easier to understand when each item is divided into categories. |
|  | I usually ensure that the staff respects the dignity of patients and families. | Considering care methods and planning individualized care into practice is important. | We need to work across facilities to create systems to protect patients. |
|  | Analysis of self-removal of tubes and falls related to physical restraints is need. | Assessment of minimization of physical restraints should include patient satisfaction survey. | Considering the best practice for minimizing physical restraints by physicians is a meaningful function for nurse executives. |
|  | Ethical considerations from the perspective of physical restraint can easily fall into a two-sided consideration of “I did not use physical restraint” and “I would inevitably use physical restraint.” | Participation in decision support by nurses is affective for minimizing physical restraints. | Although included contents in the indicators, the assessment including patients' intentions to leave beds and pull tubes that is important and should be shared and rooted. |
|  | It is useful to have a system in place to manage ethical behavior of the organization and education for training nursing leaders to build consensus on ethical issues and develop the ability to coordinate. | I thought that most of the necessary items were covered. |  |
|  | Training for preventing delirium and high skill for geriatric care is important to minimize physical restraints. | Establishing support for business improvement initiatives is important. |  |
|  | Education for preventing delirium and skill for geriatric care is important to minimize physical restraints. |  |  |

# S2 Table 4. Comments from Expert Panels for Middle Management Indicators in the 1st to 3rd Rounds

|  | **1st round modified indicator (n = 23)** | **2nd round modified indicator (n = 13)** | **3rd round modified indicator (n = 13)** |
| --- | --- | --- | --- |
| **Planning** | | | |
| 1 | Unsure whether the organization refers to hospitals, nursing departments, or wards. |  |  |
| 2 |  |  |  |
| 3 | Physical restraints might be used by taking advantage of standards determining the need for nursing using physical restraints. This role is that of the dementia care team, and not the job of head nurses. |  |  |
| 4 | Discussions are important among multidisciplinary in committees to review the need for appropriate physical restraints. |  |  |
| 5 | The committee provides feedback to the departments. | “Striving to participate in discussions” and “understanding the results of these discussions” is more suitable. The physicians could be in favor of physical restraint. Ultimately, the nurses attach and remove the physical restraint. |  |
| **Motivating** | | | |
| 6 | “Admitting staff’s positive attitude” is unclear. Evaluation? Feedback? |  |  |
| 7 |  | “Feedback recognizing the good points and autonomy of efforts” is more suitable, |  |
| 8 |  | Positive feedback is effective. |  |
| 9 |  |  |  |
| 10 | It is good if successful experiences could be shared among staff members rather than by head nurses. |  |  |
| **Training** | | | |
| 11 | I interpreted this indicator as the correct knowledge not to accept physical restraints. | I think “not to accept physical restraints” is inappropriate. The word “not accept” delegitimizes nurses. |  |
| 12 | It is important to have a demeanor that promotes calmness in patients. |  |  |
| 13 |  |  |  |
| 14 |  | Reflecting on daily ethics and usual nursing practice is important. However, it is difficult for managers to get staff to reflect on daily practice. Managers provide opportunities and staff are objective daily ethics and usual nursing practice is more effective. |  |
| 15 |  | Without support from the entire organization, research-based practice is a burden on staff. It is realistically easy to work on a practice report about a successful case.  Nurse managers should actively engage in research-based practice if they would recommend it to staff. It is effective if the study reports outcomes related to physical restraints. |  |
| **Commanding** | | | |
| 16 |  |  |  |
| 17 | I cannot evaluate because of restricted visitation owing to current COVID-19 infections. |  |  |
| 18 |  |  |  |
| 19 |  |  |  |
| 20 |  |  |  |
| 21 | It is not necessary for head nurses to directly care for patients with staff. |  |  |
| 22 | There are no guidelines for minimizing physical restraints based on evidence in Japan. |  |  |
| 23 |  |  |  |
| **Organizing** | | | |
| 24 |  | It is better if there is more than one staff member to promote the activity. |  |
| 25 | “Release” may be more suitable than “removing.” It is not enough to remove physical restraints, but the involvement needs to be tailored to the patient’s situation. We need to consider ways to prevent adopting physical restraint measures rather than whether we remove them from patients. | It does not necessarily have to be “mainly by department leaders.” |  |
| 26 |  | Brief reflections should be made to reduce the burden of the incident. | This item should include content such as “responding to prevent individual fears and accidents through team discussion.” |
| 27 | This is the function of medical safety teams and not that of head nurses. | Maintenance is important, but care must be taken not to encourage the use of physical restraints. What is the meaning of “substitutes for restraint?” |  |
| **Controlling** | | | |
| 28 | Survey and analysis is necessary, but whether this translates into nursing practice needs to be considered. This is the function of the dementia care team and not that of head nurses. |  |  |
| **Delete indicators** | | | |
| 29 | It is uncertain whether this item is the duty of the head nurse. |  |  |
| 30 | It is better to include “minimizing.” I cannot imagine this item in concrete terms. I think the expression overlaps with Nos. 12 and 13. Education that includes other professions is better. |  |  |
| 31 | Promoting initiatives by leaders and staff members in the department is suitable. |  |  |
| 32 | Survey and analysis is necessary, but whether this translates into nursing practice needs to be considered. This is the function of the dementia care team.  It should be carried out by the whole organization rather than by a particular department. |  |  |
| 33 | Survey and analysis is necessary, but whether this translates into nursing practice needs to be considered. This is the function of the dementia care team. It should be carried out by the whole organization rather than by a particular department. It is better to focus on individual patient outcomes. |  |  |
| 34 | Survey and analysis is necessary, but whether this translates into nursing practice needs to be considered. This is the function of the dementia care team. |  |  |
| **Free comment** | | | |
|  | When working with physicians based on the characteristics of the department, the head nurses can smoothly manage the department. | In addition to recommending training for staff, I would like to see the head nurses themself present an attitude of continuous learning and exploration. Head nurses should coordinate staff workloads to allow time for research and education. | These indicators are right; request for reconsideration of No. 26. |
|  | It is important to have organizational flexibility and coordination that allows for an environment that avoids physical restraints. | As a ward manager, it is necessary to have an attitude of “physical restraints will never be used.” However, in consideration of the staff’s position, there could be times when physical restraints are accepted. | No. 30 should include a survey and an analysis about accidents that occur during physical restraints.. |
|  | It is important to adjust the work system to allow staff for watching over patients as an alternative to physical restraints. | Minimizing physical restraints is a result of nursing practice. It is good to have indicators to evaluate whether environment adjustment, assessment, and care practices by staff nurses are being appropriately implemented. | In most cases, only nurses consider minimizing physical restraints. This can be realized only with the cooperation of other professions. I do not think that this indicator should be considered only by nurses. |
|  | I thought the effectiveness of each indicator varied depending on the management status, perception, and culture of physical restraints in the department. I am happy when nurse managers practice at least one of the indicators. | It is good if we could create a climate that minimizes physical restraints not only at the time of admission but also in the emergency department. This is because patients could have already been restrained in the emergency department prior to admission. | It is important to ensure that those involved do not feel responsible for accidents that could have been prevented by restraining the patient. |
|  | Head nurses need to demonstrate a willingness not to blame others for adverse incidents and accidents and to work together to resolve them and create a positive organizational culture. | These indicators are appropriate. | If the head nurse could indicate to the staff what kind of nursing practice and ethical view will be promoted as a departmental goal, it would help to organize the direction of the staff’s efforts. I think it is difficult to promote initiatives with the mindset of following the goals of the nursing department. |
|  | The nurse manager should propose goals, maintain an unwavering attitude to implementing and evaluating the plan to minimize physical restraints, and continue to provide positive feedback while acknowledging staff practices. | It is also important to understand that some physical restraints are unavoidable and not to put unnecessary pressure on staff to remove physical restraints. As in No. 2, it is important to recognize that the goal is not to achieve zero physical restraints but to minimize them. | Head nurses should support staff nurses in not feeling like they have no choice of physical restraints and practice nursing care from an ethical point of view. |
|  | Opportunity for training in delirium and dementia care has an impact on minimizing physical restraints. | It is good to create a forum in which everyone can discuss patient care and ward operations, regardless of physical restraints. | There was no discomfort for the indicators. It is better to rearrange the order of indicators by themes. |
|  | The nurse manager’s job should be to ensure that the decisions made by the dementia care team are known and thoroughly understood in the wards, and to monitor and provide feedback on any changes in staff behavior. |  |  |
|  | While treatment is the first priority, it is necessary to create a forum in which discussions with the physician can consider modified treatment to minimize physical restraints. |  |  |
|  | Changes in nurses’ attitudes toward physical restraint, including the ethical aspects, are important. |  |  |
|  | Eliminate the culture in which physical restraints are normalized. |  |  |
|  | The head nurse can create a situation in which each nurse considers what they can do for the patient. Creating an environment in which practitioners are challenged is necessary. |  |  |
|  | Physical assessment to determine the cause of the patient’s agitation or delirium and environmental adjustments to keep the patient calm are important. |  |  |
|  | It is important for head nurses to think positively about removing physical restraints. I believe that departments that do not have the opportunities to discuss physical restraints have a high number of physical restraints. |  |  |
