# Supplementary material for: Development of management indicators of nursing for minimizing physical restraints focused on older adult patients hospitalized in acute care settings: A Delphi consensus study
Source: PLoS One. 2024 Jul 10;19(7):e0306920. doi: 10.1371/journal.pone.0306920 (PMC11236117; doi:10.1371/journal.pone.0306920)
Supplement: S2 Appendix — (PDF) [file pone.0306920.s003.pdf]

Development of management indicators of nursing for minimizing physical restraints focused on older adult patients hospitalized in acute care settings: a Delphi consensus study

Survey Form (Draft Indicators for Head Nurses in middle management position)

1 Research Participation

If you understand the explanations in the attached "Request for Research Participation and Cooperation" and are willing to give your consent to participate in the study,

Please check ☑ in the "Consent to Participate in Research" box below and then answer the following questions.

The terms used here are as follows

- Older adult: Age 65 and older
- Physical restraint: Restriction of a subject's freedom of movement of part or all of the subject's body by means of restraint devices.

I agree to participate in the study ☐

2 I would like to ask you a few questions about yourself.

(1) What is your age?

1. 29 years old or younger 2. 30 years old 3. 40 years old 4. 50 years old or older

(2) Please put a circle next to your current position.

1. nurse (staff) 2. head nurse 3. assistant nurse executives 4. nurse executives 5. other

3) We ask this question only if you selected 2., 3., or 4. in 2).

How many years of experience do you have as a nursing manager?

*Nursing managers are the head nurse, assistant nurse executives, and nurse executives.

☐ years

4) We ask this question only if you selected 2., 3., or 4. in 2).

Please check all Certified Nursing Manager education course levels in Japan you have attended.

1. first level 2. second level 3. third level

5) Please put a circle in the appropriate box for your final education.

1. high school 2. technical school 3. junior college 4. university 5. graduate school

6) What is your total number of years of nursing experience? *Include non-clinical years and maternity/paternity leave.

☐ years

7) How many years of experience do you have in an acute care hospital?

☐ years

8) Please put a circle next to the nursing specialty certifications you hold.

1. certified nursing manager 2. geriatric nurse specialist 3. other ( )

3 For the following items (1) through (33), please put a "〇" in one number that you think best applies to the nursing management to be implemented by ward managers to achieve nursing practice that minimizes physical restraints for older adult patients in acute care hospitals.

		Completely invalid. ... Completely valid.	To each item Comment
1)	Visualization of issues related to physical restraints in the organization	1・2・3・4・5・6・7・8・9	
2)	Developing departmental targets to understand the nursing department's policy to minimize physical restraints	1・2・3・4・5・6・7・8・9	
3)	Documenting standards for staff to determine the need for physical restraints	1・2・3・4・5・6・7・8・9	
4)	Grasping the status of efforts to minimize physical restraints	1・2・3・4・5・6・7・8・9	
5)	Encouraging staff to take advantage of problem-solving opportunities with multidisciplinary to minimize physical restraints	1・2・3・4・5・6・7・8・9	
6)	Admitting staff's positive attitude toward minimizing physical restraints	1・2・3・4・5・6・7・8・9	
7)	Feedback on what is discussed about physical restraints at committee meetings and in administrative departments for staff	1・2・3・4・5・6・7・8・9	
8)	Improving staff self-efficacy by sharing patient/family responses to staff who have implemented nursing care to minimize physical restraints	1・2・3・4・5・6・7・8・9	
9)	Sharing success experiences of minimizing physical restraints	1・2・3・4・5・6・7・8・9	
10)	Providing opportunities or encouraging participation in education to spread the correct knowledge and skills about physical restraints	1・2・3・4・5・6・7・8・9	
11)	Providing opportunities or encouraging participation in	1・2・3・4・5・6・7・8・9	

		Completely **invalid.** ... Completely **valid.**	To each item Comment
	education as a base for older adult patients’ care and delirium care		
12)	Enabling reflection on nursing care from the patient’s point of view to foster an ethical view of physical restraints	1・2・3・4・5・6・7・8・9	
13)	Enhancing to reflect on usual nursing practice from the perspective of ethics	1・2・3・4・5・6・7・8・9	
14)	Research practice to minimize physical restraints	1・2・3・4・5・6・7・8・9	
15)	Setting up a discussion forum for multiple staff members in the department to discuss minimizing physical restraints	1・2・3・4・5・6・7・8・9	
16)	Providing opportunities for dialogue with patients and families regarding physical restraints to gain their understanding and cooperation in minimizing physical restraints	1・2・3・4・5・6・7・8・9	
17)	Considering care methods for factors that contribute to physical restraints with cross-functional teams and specialists	1・2・3・4・5・6・7・8・9	
18)	Discussing with other professionals and specialists about ethical dilemmas regarding physical restraints	1・2・3・4・5・6・7・8・9	
19)	Sharing ethical issues related to physical restraints with staff	1・2・3・4・5・6・7・8・9	
20)	Collaborating with staff to determine care alternatives to physical restraints in the field	1・2・3・4・5・6・7・8・9	
21)	Use of manuals and guidelines to address factors of physical restraints adopted	1・2・3・4・5・6・7・8・9	

		Completely invalid. … Completely valid.	To each item Comment
22)	Negotiation with administrative departments on providing necessary environmental arrangements to minimize physical restraints	1・2・3・4・5・6・7・8・9	
23)	Supporting the activities of staff that promote minimizing physical restraints	1・2・3・4・5・6・7・8・9	
24)	Trying to discuss the point of removal of physical restraints	1・2・3・4・5・6・7・8・9	
25)	Responding to reduce fear about accidents associated with minimizing physical restraint with an attitude of accepting responsibility	1・2・3・4・5・6・7・8・9	
26)	Prepare the accident prevention items and restraint substitutes	1・2・3・4・5・6・7・8・9	
27)	Survey and analysis of the number and percentage of physical restraints	1・2・3・4・5・6・7・8・9	
28)	Communicating to staff the policy of minimizing physical restraints	1・2・3・4・5・6・7・8・9	
29)	Recommending the use of educational tools to ensure that all staff receive education on physical restraints	1・2・3・4・5・6・7・8・9	
30)	Involving the chief and specialists to facilitate so that staff can discuss care of minimizing physical restraints	1・2・3・4・5・6・7・8・9	
31)	Survey and analysis of staff's awareness of physical restraints	1・2・3・4・5・6・7・8・9	
32)	Analysis of physical restraint rates with comparing to external evaluation criteria	1・2・3・4・5・6・7・8・9	
33)	Reflecting for practice from the results of the survey analysis for physical restraints	1・2・3・4・5・6・7・8・9	

4 In addition to the above, please list below the indicator items that you believe are necessary for nursing management to achieve nursing practice that minimizes physical restraints for older adult patients in acute care hospitals.

This concludes all surveys. Thank you for your cooperation.
