# Supplementary material for: Development of management indicators of nursing for minimizing physical restraints focused on older adult patients hospitalized in acute care settings: A Delphi consensus study
Source: PLoS One. 2024 Jul 10;19(7):e0306920. doi: 10.1371/journal.pone.0306920 (PMC11236117; doi:10.1371/journal.pone.0306920)
Supplement: S3 Appendix — (PDF) [file pone.0306920.s004.pdf]

Development of management indicators of nursing for minimizing physical restraints focused on older adult patients hospitalized in acute care settings: a Delphi consensus study

Survey Form

(Draft Indicators for nurse executives and assistant nurse executives in top management position)

1 Research Participation

If you understand the explanations in the attached "Request for Research Participation and Cooperation" and are willing to give your consent to participate in the study,

Please check ☑ in the "Consent to Participate in Research" box below and then answer the following questions.

The terms used here are as follows

- Older adult: Age 65 and older
- Physical restraint: Restriction of a subject's freedom of movement of part or all of the subject's body by means of restraint devices.

I agree to participate in the study ☐

2 I would like to ask you a few questions about yourself.

(1) What is your age?

1. 29 years old or younger 2. 30 years old 3. 40 years old 4. 50 years old or older

(2) Please put a circle next to your current position.

1. nurse (staff) 2. head nurse 3. assistant nurse executives 4. nurse executives 5. other

3) We ask this question only if you selected 2., 3., or 4. in 2).

How many years of experience do you have as a nursing manager?

*Nursing managers are the head nurse, assistant nurse executives, and nurse executives.

☐ years

4) We ask this question only if you selected 2., 3., or 4. in 2).

Please check all Certified Nursing Manager education course levels in Japan you have attended.

1. first level 2. second level 3. third level

5) Please put a circle in the appropriate box for your final education.

1. high school 2. technical school 3. junior college 4. university 5. graduate school

6) What is your total number of years of nursing experience? *Include non-clinical years and maternity/paternity leave.

☐ years

7) How many years of experience do you have in an acute care hospital?

☐ years

8) Please put a circle next to the nursing specialty certifications you hold.

1. certified nursing manager 2. geriatric nurse specialist 3. other ( )

3 For the following items (1) through (35), please put a "O" in one number that you think <u>best applies</u> to the nursing management to be implemented by nurse executives and assistant nurse executives to achieve nursing practice that minimizes physical restraints for older adult patients in acute care hospitals.

		Completely **invalid.** … Completely **valid.**	To each item Comment
1)	Incorporating findings on minimizing physical restraints outside of hospitals and on the environment surrounding acute care hospitals	1・2・3・4・5・6・7・8・9	
2)	Visualization of issues related to physical restraints occurring in the nursing department	1・2・3・4・5・6・7・8・9	
3)	Statement of policy to minimize physical restraints	1・2・3・4・5・6・7・8・9	
4)	Instilling the policy of minimizing physical restraints at the nursing department	1・2・3・4・5・6・7・8・9	
5)	Clarification on the definition of physical restraint	1・2・3・4・5・6・7・8・9	
6)	Providing standards in the hospital to determine the need for physical restraints	1・2・3・4・5・6・7・8・9	
7)	Creating manuals or guidelines that address the factors that lead to the implementation of physical restraints	1・2・3・4・5・6・7・8・9	
8)	Sharing success experiences of minimizing physical restraints within the nursing department	1・2・3・4・5・6・7・8・9	
9)	Sharing the reactions of patients and families to the implementation of nursing care to minimize physical restraints within the nursing department	1・2・3・4・5・6・7・8・9	
10)	Being an organizational culture to reduce physical restraints	1・2・3・4・5・6・7・8・9	
11)	Planning for required education to minimize physical restraints based on the actual situation and existing evidence of physical restraints	1・2・3・4・5・6・7・8・9	
12)	Assigning staff in charge of	1・2・3・4・5・6・7・8・9	

		Completely **invalid.** … Completely **valid.**	To each item Comment
	promoting the minimization of physical restraints at each department		
13)	Educating the staff to spread the correct knowledge and skills necessary for minimizing physical restraints	1・2・3・4・5・6・7・8・9	
14)	Educating the staff to acquire skills to minimize physical restraints	1・2・3・4・5・6・7・8・9	
15)	Researching efforts to minimize physical restraints	1・2・3・4・5・6・7・8・9	
16)	Adopting educational tools to ensure that all staff members receive education related to minimizing physical restraints	1・2・3・4・5・6・7・8・9	
17)	Encouraging staff voluntary growth and helping it reflect on daily ethics and usual nursing practice regarding physical restraints from an ethical perspective	1・2・3・4・5・6・7・8・9	
18)	Survey and analysis of staff' s awareness of physical restraints	1・2・3・4・5・6・7・8・9	
19)	Sharing with each department the discussions among managers and committees regarding physical restraints to review ethics aspect	1・2・3・4・5・6・7・8・9	
20)	Intentionally involving individuals in a leadership position in the department: the head nurse and chief nurse	1・2・3・4・5・6・7・8・9	
21)	Considering the impact of no use of physical restraints on hospital management and sharing with other departments and nursing departments	1・2・3・4・5・6・7・8・9	
22)	Placing cross-organizational teams to promote minimizing physical restraints	1・2・3・4・5・6・7・8・9	

		Completely **invalid.** … Completely **valid.**	To each item Comment
23)	Making efforts to minimize physical restraints by multidisciplinary teams or multiple staff	1・2・3・4・5・6・7・8・9	
24)	Considering care methods to cooperate with staff in each department and cross-sectional teams	1・2・3・4・5・6・7・8・9	
25)	Creating a system to consult with specialists in the hospital regarding physical restraints	1・2・3・4・5・6・7・8・9	
26)	Establishing a system to share ethical issues related to physical restraints with managers, each committee, and each medical department	1・2・3・4・5・6・7・8・9	
27)	Devising a work system to look after patients	1・2・3・4・5・6・7・8・9	
28)	Showing attitude to guarantee responsibility against accidents associated with minimizing physical restraints	1・2・3・4・5・6・7・8・9	
29)	Creating an environment, maintenance, and management of supplies to prevent accidents	1・2・3・4・5・6・7・8・9	
30)	Ongoing evaluation of the efforts progress minimizing physical restraints at your hospital	1・2・3・4・5・6・7・8・9	
31)	Survey and analysis of the number and percentage of physical restraints in the hospital	1・2・3・4・5・6・7・8・9	
32)	Analysis of physical restraint rates by comparing with external evaluation criteria	1・2・3・4・5・6・7・8・9	
33)	Reflecting for practice from the results of the survey analysis for physical restraints	1・2・3・4・5・6・7・8・9	
34)	Placing a committee to review issues related to physical restraints	1・2・3・4・5・6・7・8・9	

		Completely **invalid**. … Completely **valid**.	To each item Comment
35)	Creating opportunities to reflect on nursing practices from the patient's perspective to foster an ethical view of physical restraints	1 ・ 2 ・ 3 ・ 4 ・ 5 ・ 6 ・ 7 ・ 8 ・ 9	

4 In addition to the above, please list below the indicator items that you believe are necessary for nursing management to achieve nursing practice that minimizes physical restraints for older adult patients in acute care hospitals.

This concludes all surveys. Thank you for your cooperation.
