# Supplementary material for: Development of management indicators of nursing for minimizing physical restraints focused on older adult patients hospitalized in acute care settings: A Delphi consensus study
Source: PLoS One. 2024 Jul 10;19(7):e0306920. doi: 10.1371/journal.pone.0306920 (PMC11236117; doi:10.1371/journal.pone.0306920)
Supplement: Attachment — Submitted filename: Manuscript number PONE-D-23-31249.pdf [file pone.0306920.s005.pdf]

Development of management indicators of nursing for minimizing physical restraints focused on older adult patients hospitalized in acute care settings: a Delphi consensus study

Comments:

It is significant, valuable study, as it addresses an important and ethical issues in missed area (old age care). This study will contribute to fill the gap of knowledge related using of constrain and solving the real problem encounter nurses caring for elderly in acute care setting as well as in geriatric home.

Abstract: page no 2: Revise the numbers of items or indicators allocated for the middle management and matched it with the rest of study.

Introduction:

- It required to provides background information on the topic.
- It is very concise and it is preferable to write a little about role of nurse's advocacy as it important in reducing using of restrain particularly among vulnerable frail elderly.
- Add reference to the paragraph no 53

Materials and methods:

- Expand a little and describes the methods used to obtain the codes from literature.
- Use the term indicators instead of (codes) or vis versa: as it preferable to use the same term.

Participants:

- In paragraph 105 add justification why select convenience technique, although the participant frame work is well known.
- Clearly state inclusion and exclusion criteria for participant

Analysis:

- Add the finding of RAND/UCLA Appropriateness Method
- Researcher need to perform the reliability test: to use these indicators to minimize physical restraints again

Ethics: Attach the ethical approval document.

Table 1. Participant Characteristics 174

- Revise the categories of participants in table 1: staff nurses present in this table, and in the rest of study there are two categories: Middle management position and top management position

Discussion: Well written**,** precise and concise

Limitations. 280: Well written, but the researcher needs to add sampling technique used as one of limitations

Conclusion.288

Rewrite it again: Summarizes the main points and suggests implications for future research.

References.302: Update the old references: it is preferable not to use a reference before 2013

Note: - Reduce the use of AI as it reached 45%

At the end: This research is considering a real addition to the body of knowledge in geriatric nursing field

With my best wishes for success

Nahla Elradhi
